# In Silico Analysis of Ferroptosis-Related Genes and Its Implication in Drug Prediction against Fluorosis

**DOI:** 10.3390/ijms24044221

**Published:** 2023-02-20

**Authors:** Bin Liu, Xiaoli Fu, Yuhui Du, Zichen Feng, Xiaoxue Liu, Zhiyuan Li, Fangfang Yu, Guoyu Zhou, Yue Ba

**Affiliations:** Department of Environmental Health, School of Public Health, Zhengzhou University, Zhengzhou 450001, China

**Keywords:** ferroptosis, fluorosis, molecular docking, molecular dynamics simulation, therapeutic drugs

## Abstract

Fluorosis is a serious global public health problem. Interestingly, so far, there is no specific drug treatment for the treatment of fluorosis. In this paper, the potential mechanisms of 35 ferroptosis-related genes in U87 glial cells exposed to fluoride were explored by bioinformatics methods. Significantly, these genes are involved in oxidative stress, ferroptosis, and decanoate CoA ligase activity. Ten pivotal genes were found by the Maximal Clique Centrality (MCC) algorithm. Furthermore, according to the Connectivity Map (CMap) and the Comparative Toxicogenomics Database (CTD), 10 possible drugs for fluorosis were predicted and screened, and a drug target ferroptosis-related gene network was constructed. Molecular docking was used to study the interaction between small molecule compounds and target proteins. Molecular dynamics (MD) simulation results show that the structure of the Celestrol–HMOX1 composite is stable and the docking effect is the best. In general, Celastrol and LDN-193189 may target ferroptosis-related genes to alleviate the symptoms of fluorosis, which may be effective candidate drugs for the treatment of fluorosis.

## 1. Introduction

Fluorine is widely distributed in nature. As an extremely active element with chemical properties, most of it exists in water, rock, and soil in the form of fluoride [1]. In humans, fluoride can enter the body through drinking water, diet, and air. In organisms, fluoride in organisms can be deposited in calcified tissues to prevent dental caries and strengthen bones [2]. Concerning public safety, the World Health Organization(WHO) has established the safe limit of fluoride intake at 1.5 parts per million (PPM) [3]. This indicates that the permissible limit of fluoride intake is stricter compared to the other essential trace elements. With some adverse effects, excessive intake of fluoride or long-term exposure to high fluoride will increase the metabolic burden of the body. Additionally, excessive fluoride accumulation in various tissues and organs of the body will lead to varying degrees of damage [4]. In brief, a large number of studies have confirmed that excessive fluoride intake mainly causes dental fluorosis and skeletal fluorosis [5,6]. That is why, in recent years, the toxic effects of fluoride on soft tissue—including the liver, kidney, and brain—have gradually attracted attention, and especially the toxic effects of fluoride exposure on the nervous system and reproductive system [7,8,9,10]. Specifically, in the 19th and 20th centuries, fluorosis was regarded as an incurable disease [11]. Nevertheless, a combination of safe drinking water, nutritional supplements, and health services has recently improved the prevalence of skeletal fluorosis both in adults and children under 12 years of age [3]. Particularly, a meta-analysis conducted in a fluorosis region in China confirmed that water quality improvement and defluorination significantly reduced the prevalence of skeletal fluorosis and urinary fluoride levels in adults [12], but so far, there is no effective drug for the treatment of fluorosis.

Ferroptosis was first discovered in 2012 as a unique form of cell death induced by the small-molecule compound called erastin [13]. Ferroptosis is caused by the inactivation of antioxidant defense dependent on glutathione (GSH) in cells, resulting in excessive peroxidation of the phospholipid (PL) membrane rich in polyunsaturated fatty acids (PUFAs), which in turn causes the accumulation of toxic lipid ROS (l-ROS), and then leads to cell death through an iron-dependent mechanism [14,15]. Ferroptosis cells have typical necrotic morphology, accompanied by small abnormal mitochondria, reduced cristae, membrane condensation, rupture of the outer membrane, and no apoptotic characteristics [16,17,18]. A large number of studies indicate that fluoride can induce oxidative stress by increasing the levels of reactive oxygen species (ROS) and malondialdehyde (MDA), and further lead to ferroptosis [19,20]. The discovery of ferroptosis in fluorosis suggests that ferroptosis is a new mechanism of fluorosis, and the targeted intervention of ferroptosis is expected to be an effective method for the treatment of fluorosis.

In this study, we used bioinformatics and multiple public databases to screen important ferroptosis-related genes and pathways of fluorosis, expecting to find potential therapeutic drugs and provide clues for further prevention and follow-up treatment of fluorosis.

## 2. Result

### 2.1. Identification of Differential Genes

**Figure 1:**
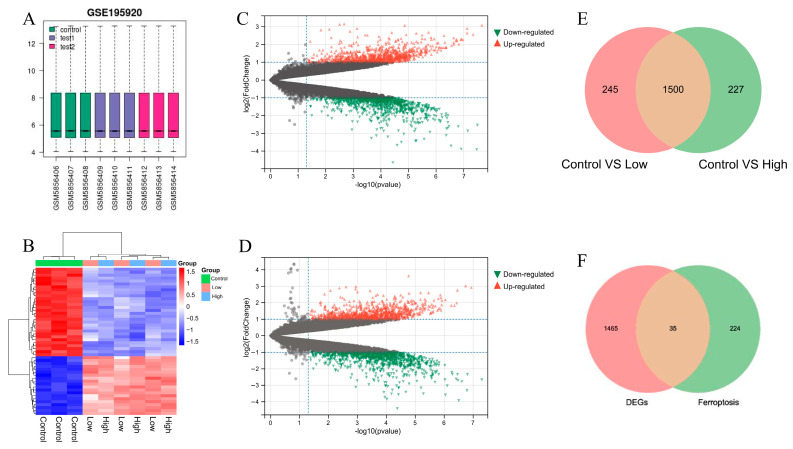
Expression profile of differentially expressed mRNA after fluoride exposure. (**A**) Comparability evaluation of microarray data. (**B**) Heat map of the top 50 DEGs (red: high expression; blue: low expression). (**C**,**D**) Distribution of DEGs in the volcanic map (**C**) control vs. low; (**D**) control vs. high). (**E**) Venn map of differential genes. (**F**) Venn map of ferroptosis-related genes.

The GSE195920 gene data set consisted of the control groups, the 0.095 μg/mL fluoride exposure group (low) and the 0.22 μg/mL fluoride exposure group (high). Using the “limma” package for differential expression analysis, it was found that 1745 genes in the 0.095 μg/mL fluoride exposed group were differentially expressed compared with the normal group, where 874 were upregulated and 871 were downregulated. As expected, 1727 genes in the 0.22 μg/mL fluoride exposure group were differentially expressed compared with those in the normal group, among which 828 were upregulated and 899 were downregulated. Taking the intersection between the two, a total of 1500 differential genes were obtained (Figure 1E), including 732 upregulated genes and 768 downregulated genes. Significantly, the data were analyzed for standardization and comparability. As shown in Figure 1A, the selected sample distribution reached the expected standard, indicating high quality and comparability of the microarray data. Further, cluster analysis was performed on these differential genes and a volcanic map was drawn (Figure 1C, D). The first 50 differential expressions were those considered and presented through a heat map (Figure 1B).

### 2.2. Overlap of DEGs and Ferroptosis-Related Genes and Venn Analysis

We overlapped the DEGs with ferroptosis-related genes extracted from the FerrDb. A total of 35 overlapping genes were obtained, as shown in Figure 1F. The expression level is shown in the attached Table S1. Details of overlapping genes, including 16 drivers, 15 suppressors, and 15 markers, are presented in Table 1. We also observed that some genes play different roles.

### 2.3. Gene Ontology (GO) and KEGG Pathway Analysis of Overlapping Genes

**Figure 2:**
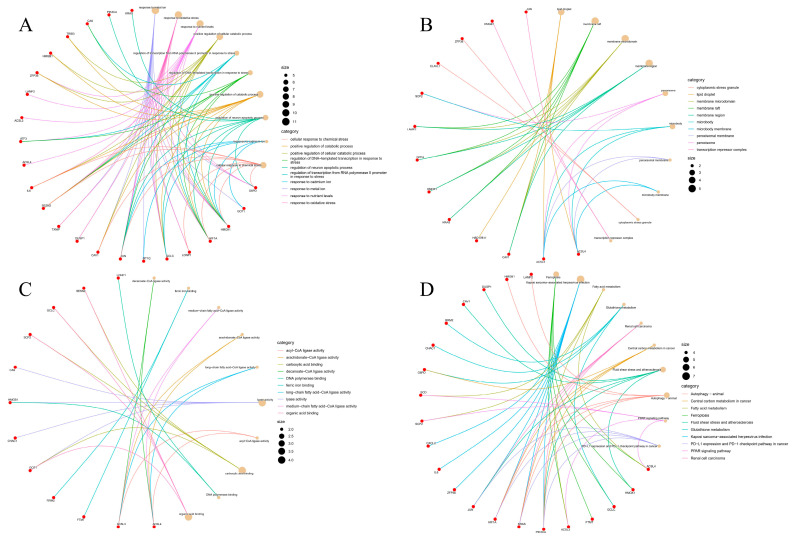
Analysis of differentially expressed mRNA (DEGs) enriched by GO and KEGG pathways. (**A**) Top ten MF analysis. (**B**) Top ten CC analysis. (**C**) Top ten MF analysis. (**D**) The bubble chart shows the KEGG pathway in the top 10 DEGs.

To clarify the biological function and the pathway related to the 35 DEGs, GO enrichment and KEGG pathway analysis were carried out. GO analysis (Figure 2A) confirmed that the 35 DEGs were mainly involved in biological processes such as response to the metal ion, oxidative stress, and nutrient levels. Figure 2B shows that the genes are mainly located in lipid droplets, membrane raft, membrane microdomain, etc. Functional analysis shows that the main molecular functions of the 35 DEGs are decanoate CoA ligase activity, ferric iron binding, medium-chain fat acid CoA ligase activity, etc. (Figure 2C). Next, we performed the KEGG pathway analysis, which further suggested that the 35 DEGs are mainly involved in ferroptosis, Kaposi sarcoma-associated herpesvirus infection, fatty acid metabolism, glutathione metabolism, renal cell cancer, and other pathways (Figure 2D).

### 2.4. PPI Network Construction and Hub Gene Screening

**Figure 3:**
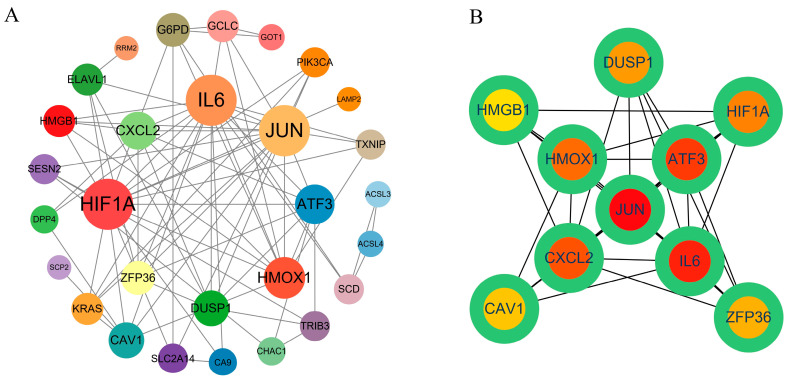
Construction of PPI network and hub gene screening. (**A**) PPI network constructed by string database and Cytoscape visualization. (**B**) Hub gene identified by Cytoscape based on MCC algorithm.

To further explore the role of the 35 DEGs associated with ferroptosis, with a PPI analysis of the 35 DEGs ferroptosis-related differential genes in the STRING database, a network containing 33 nodes and 80 sides of fluorine exposure was constructed. For visualization purposes, Cytoscape v3 9.1 was employed (Figure 3A). Additionally, the MCC algorithm of the cytohubba plug-in was used to identify the hub gene, and 10 hub genes were isolated from the 35 overlapping genes, and ranked from high to low as follows: Jun, IL6, ATF3, cxcl2, HMOX1, HIF1A, DUSP1, zfp36, cav1, and HMGB1 (Figure 3B).

### 2.5. Screening of Potential Pharmacological Targets

To explore the potential molecular drugs of fluorosis, we obtained the screening results of the potential pharmacological targets from the CMap and the classification was performed according to the drug connectivity score. The top 10 drugs have been reported in previous studies, namely Celastrol [21], LDN-193189 [22], XPNPEP1 [23], GNB5 [24], Importazole [25], LDHB [26], NUAK1 [27], IFNG [28], IFNB1 [29], and YWHAH [30], suggesting that these drugs may be effective candidate drugs for the treatment of fluorosis (Table 2).

### 2.6. Validation of Potential Therapeutics against CTD Database

**Figure 4:**
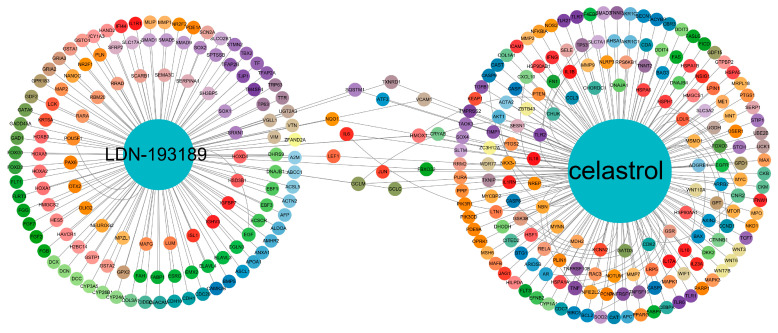
Construction of predicted drug–target gene interaction network.

Target gene prediction for the above 10 potential therapeutic drugs shows that Celastrol has 375 target genes and 4 overlapping genes (JUN, IL6, ATF3, and HMOX1), as revealed by the intersection of the 10 hub genes. Similarly, LDN-193189 predicted 138 target genes, intersected with 10 hub genes, and found 1 overlapping gene (HMOX1). The other eight drugs had no predicted target genes (Figure 4).

### 2.7. Verification of Protein Expression of Overlapping Genes

**Figure 5:**
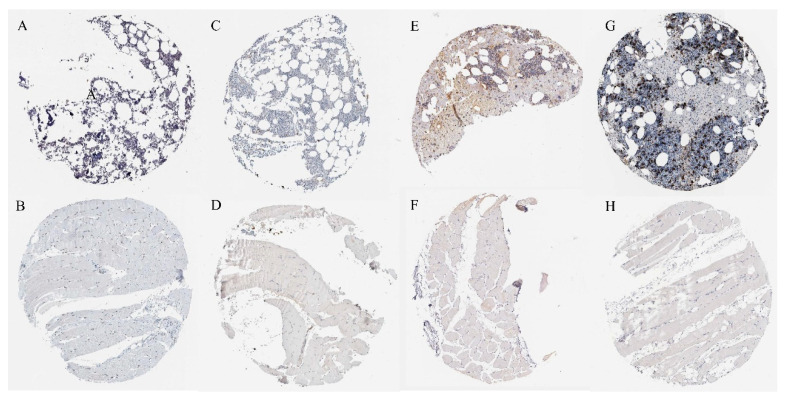
(**A**) Expression of JUN protein in the bone marrow (one of the outcomes). (**B**) Expression of JUN protein in skeletal muscle. (**C**) Expression of IL6 protein in the bone marrow. (**D**) Expression of IL6 protein in skeletal muscle. (**E**) Expression of ATF3 protein in the bone marrow. (**F**) Expression of ATF3 protein in skeletal muscle. (**G**) Expression of HMOX1 protein in the bone marrow. (**H**) Expression of HMOX1 protein in skeletal muscle.

To verify the protein expression of four overlapping genes (JUN, IL6, ATF3, and HMOX1), we searched for the expression of four overlapping genes in the bone marrow and skeletal muscle based on the HPA database (Figure 5). Immunohistochemical results showed that compared with skeletal muscle, the protein expression of the four overlapping genes was highly expressed in the bone marrow. Our previous transcriptome analysis showed that fluoride exposure reduced the expression of those four overlapping genes, which was further verified by proteomics.

### 2.8. Molecular Docking Reveals Potential Targets for Small-Molecule Drugs and Proteins

**Figure 6:**
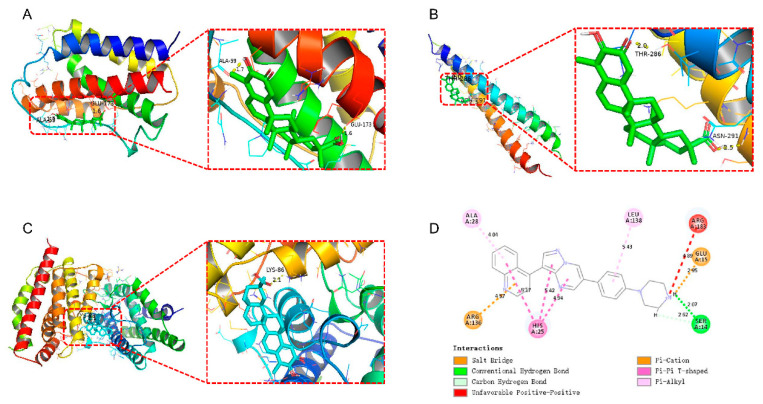
Molecular docking interaction map. (**A**) Celastrol–IL6 complex. (**B**) Celastrol–JUN complex. (**C**) Celastrol–HMOX1 complex. (**D**) LDN-193189–HMOX1 complex. Among the interactions, green represents van der Waals interactions, light green represents carbon–hydrogen bonds, red represents unfavorable Positive–Positive, yellow represents salt bridges and π-cations, and pink represents alkyl and π–alkyl interactions.

To further understand the potential targets of the screened small-molecule drugs and proteins, we conducted molecular docking studies. As shown in Figure 6A, the results revealed that Celastrol can form hydrogen bonds with ALA-59 and GLU-173 (amino acid residues) of IL6 (PDB ID: 1IL6), with a known binding energy of 11 kcal/mol and corresponding bond lengths of 1.7 Å and 1.6 Å, respectively. Additionally, Celastrol can form hydrogen bonds with THR-286 and ASN-291 (amino acid residues) of JUN (PDB ID: 1JUN), with an involved binding energy of −6.8 kcal/mol and resultant bond lengths of 2.0 Å and 2.5 Å, respectively (Figure 6B). Furthermore, Celastrol can form hydrogen bonds with LYS-86 (amino acid residue) of HMOX1 (PDB ID: 1N45), with a binding energy of −8.5 kcal/mol and consequent bond length of 2.1 Å (Figure 6C). In identifying the interactions between LDN-193189 and HMOX1 amino acids, Discovery Studio was used. Thereafter, we observed that the amino acids in HMOX1 that interact with LDN-193189 included ALA A:28, LEU A:138, ARG A:136, GLU A:15, and SER A:14. Moreover, the distance-dependent interactions between the two included van der Waals interactions, conventional hydrogen bonds, salt bridges, carbon–hydrogen bonds, attractive charges, alkyl bonds, and π–alkyl interactions (Figure 6D). The results of molecular docking are shown in Table S2. Unfortunately, the crystal structure of the ATF3 protein was not retrieved.

### 2.9. Molecular Dynamics Simulation

**Figure 7:**
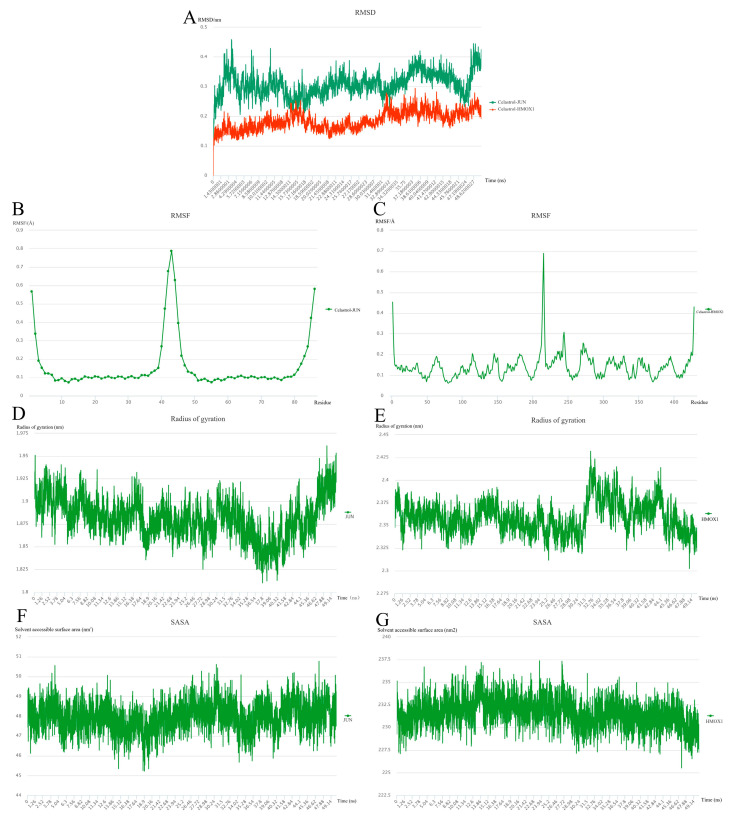
Molecular dynamics simulation for 50 ns. (**A**) RMSD of Celastrol–JUN and Celastrol–HMOX1. (**B**,**C**) The per-residue RMSF of Celastrol bound with JUN and HMOX1. (**D**,**E**) Rg of Celastrol–JUN and Celastrol–HMOX1. (**F**,**G**) The SASA of Celastrol bound with JUN and HMOX1.

MD simulation can provide more comprehensive information on drug receptor dynamics, which, combined with the above molecular docking results, can better rank small-molecule predictive drugs. MD simulations after docking can provide complete information on small molecules that predict the stability of drug–protein complexes and the structural changes associated with their binding. We performed 50 ns MD simulations of JUN or HMOX1 combined with Celastrol. The RMSD results showed that the average value of the Celestrol–HMOX1 complex was about 1.5 Å, lower than that of the Celestrol–JUN complex (Figure 7A). It shows that the combination of the Celastol–HMOX1 complex is more stable. The RMSF of the two complexes was studied to understand the flexibility of the amino acid region of JUN and HMOX1. Figure 7B,C shows that almost all residues have low RMSF. Rg was used to evaluate the compactness of protein complexes. Higher values indicate more unstable properties, while lower values indicate the robustness of the protein. As shown in Figure 7 D,E, the Rg value of HMOX1 is still decreasing in the last 5 ns, while the Rg value of JUN shows a rising trend. To evaluate the change in surface response and peptide binding, we then analyzed the SASA value of the complex. The average SASA values of Celastol–JUN and Celastol–HMOX1 complexes were 47.9786 nm^2^ and 231.6303 nm^2^, respectively (Figure 7F,G). During the whole simulation period, the SASA values of the two compounds did not fluctuate significantly, and the fluctuation range was small, indicating that the composite did not expand or contract during the simulation period [31].

## 3. Discussion

With the application of deflouridation project of drinking water and replacing stoves [32,33], the prevalence of fluorosis, especially dental and skeletal fluorosis, has been reduced greatly. However, due to the complex natural environment and the comprehensive effect of fluoride on the body, the prevalence of fluorosis is still serious [3]. Since the discovery of ferroptosis, its toxic mechanism regarded as exogenous chemicals has attracted extensive attention. However, the pathogenesis of ferroptosis in fluorosis is not clear, and the key genes and pathways related to ferroptosis in fluorosis need to be further discovered [19]. In this study, 35 differentially expressed genes (17 upregulated and 18 downregulated) related to ferroptosis in fluorosis were obtained by analyzing the gene expression profile of the fluoride exposure model for the first time and were used to indicate that ferroptosis-related genes are involved in the pathogenesis of fluorosis, suggesting potential pharmacological targets.

To understand the role of these ferroptosis-related genes in fluorosis, we further carried out GO enrichment analysis and KEGG pathway analysis. GO enrichment analysis showed that 35 DEGs were mainly involved in the response to the metal ion, oxidative stress, neuronal apoptosis, and so on. In the literature, it has been reported that NaF at different concentrations inhibited the proliferation of mouse primary osteoblasts, the expression of insulin-like growth factor-I (IGF-I), and the level of oxidative stress [34]. In mouse liver cells, NaF triggers oxidative stress and further induces apoptosis by promoting the production of reactive oxygen species and reducing antioxidant function [35]. Our recent KEGG pathway analysis found that the differential expression of ferroptosis-related genes in fluorosis is mainly involved in ferroptosis, fatty acid metabolism, and glutathione metabolism. Besides that, in a fluoride-containing medium, the degree of ferroptosis of sub-cultivating MC3T3-E1 (WT) cells was higher than that of fluoride-resistant MC3T3-E1 (FR) cells, indicating that there is a potential link between fluorosis and ferroptosis [19]. In addition, a cross-sectional study of schoolchildren reported that Glutathione S-Transferase (GST) levels were positively associated with the degree of fluorosis [36]. In verification, children with higher exposure to fluoride concentrations in saliva had higher GST activity [36]. Taken together, the existing evidence shows that inhibiting oxidative stress and targeted inhibition of ferroptosis may constitute a potential new method for the treatment of fluorosis.

By constructing the PPI network of ferroptosis-related differentially expressed genes, we screened 10 hub genes: Jun, IL6, ATF3, cxcl2, HMOX1, HIF1A, DUSP1, zfp36, cav1, and HMGB1. Significantly, these genes are mainly involved in oxidative stress, oxygen homeostasis, cell proliferation, inflammation, and immune responses. Zfp36, HMOX1, and ATF3 have antioxidant effects and play important roles in tumor progression. Zfp36, also known as tristetraprolin, has a neuroprotective effect and protects dopaminergic neurons from oxidative damage [37]. However, unlike Zfp36, HMOX1 has a dual role in tumor cells. While under-activation of HMOX1 plays a cytoprotective role by scavenging reactive oxygen species, over-activation increases the level of reactive oxygen species and iron concentration to induce apoptosis [38]. Activating transcription factor 3 (ATF3) is a member of the ATF/CREB transcription factor family. Stress responses such as DNA damage, oxidative stress, and cell damage can quickly induce its expression [39]. HIF1A and DUSP1 are closely related to oxygen homeostasis. As a subunit of HIF1, HIF1A is involved in regulating the homeostatic response of cells and systems related to oxygen [40]. Like HIF1A, DUSP1 is also a hypoxia-related gene [41], which can be induced by hypoxia, nutrient deprivation, oxidative stress, and other reactions [42]. Jun [43] and CAV1 can regulate cell proliferation, apoptosis, and differentiation and inhibit tumor angiogenesis [44]. IL-6 [45,46], HMGB1 [47], and Cxcl2 [48] play important roles in inflammatory and immune responses as soluble mediators, multifunctional proteins, and chemokines, respectively.

According to CMap analysis, we obtained 10 compounds that may be used in the treatment of fluorosis. Celastrol is a powerful bioactive natural product derived from the root bark of *Tripterygium wilfordii*, which has a variety of biological characteristics, such as anti-tumor and immunosuppressive properties [49]. It is reported that in chondrocytes, fluoride can inhibit chondrocyte proliferation by inhibiting the PI3K/Akt/mTOR signaling pathway [50]. In microglia, NaF increases the activity of GSK-3β and NF-κB, leading to the release of pro-inflammatory mediators IL-6 and TNF-α, further aggravating the inflammatory response [51]. Interestingly, studies have confirmed that Celastrol reduces the inflammatory response by enhancing the Nrf2/ho-1 pathway and the PI3K/AKT signaling pathway and inhibiting NF-κB activation [52]. Given this evidence, Celastrol has potential for the treatment of fluorosis. Fluoride can inhibit cartilage formation and proliferation [50], while LDN-193189, as a multi-cord morphoprotein derivative, is a powerful selective activin receptor-like kinase 2 (ALK2) and ALK3 inhibitor [53], which can promote cartilage formation of bone marrow stromal cells [54] and resist the damage of fluoride to the bone to a certain extent. Further, the CTD database prediction found that Celastrol could interact with four hub genes (JUN, IL6, ATF3, HMOX1), and LDN-193189 could interact with one hub gene (HMOX1). Regarding the immunohistochemical analysis, the results from the HPA database confirmed that fluoride exposure reduced the expression of four overlapping genes. Thus, targeted drugs may achieve the effect of treating fluorosis by regulating these ferroptosis-related genes. The molecular docking also revealed that triptolide interacts with amino acid residues on SLC7A11 via van der Waals forces, carbon–hydrogen bonds, and conventional hydrogen bonds. The data also supported that triptolide can directly combine with SLC7A11 to play a regulatory role [55]. As revealed in this study, molecular docking results showed that the selected small-molecule drugs could combine with ferroptosis-related protein receptors to exert therapeutic effects. To further evaluate the reliability of molecular docking results, molecular dynamics simulation was used to analyze the RMSD, RMSF, Rg, and SASA of the docking complex. The results showed that the structure of the Celestrol-HMOX1 composite was stable and the docking effect was the best. The above analysis process is detailed in flowchart S1 in the Supplementary Materials.

Fluorosis is a slow and progressive disease that affects human health. It is well known that fluoride can disrupt oxidative stress, endoplasmic reticulum stress [56], glycolysis [57], and nerve conduction [58]. Severe skeletal fluorosis is accompanied by chronic pain and even disability for a long time. Markedly, so far, there is no specific treatment [59]. In this study, we constructed the protein interaction network of ferroptosis-related genes in fluorosis for the first time and screened the targeted therapeutic drugs of fluorosis. Although it has been reported that chronic symptoms of skeletal fluorosis are irreversible and permanent [60], a double-blind controlled trial in children found that treatment with ascorbic acid, calcium, and vitamin D3 significantly improved children’s dental, clinical, and skeletal fluorosis and related biochemical indicators. This shows that fluorosis can be reversed [61]. Similarly, skeletal fluorosis can be alleviated and reversed by eliminating fluoride intake, supplementing calcium, and supplementing a diet rich in antioxidants [62,63]. Targeted therapy for the above-mentioned genes can improve the prognosis of patients with fluorosis, which is a technology with broad application prospects.

## 4. Materials and Methods

### 4.1. Source of Microarray Data

Gene Expression Omnibus (GEO, https://www.ncbi.nlm.nih.gov/geo/, accessed on 1 April 2022) is a public database from which gene expression data can be freely accessed. In this study, we downloaded a microarray data set (GSE195920) from the GEO database to investigate the effect of fluoride exposure on the gene expression profile of HOS (human osteosarcoma) cells [64]. Thereafter, the GSE195920 gene expression profile was constructed based on the GPL16699 Affymetrix human genome array platform. Then, the GSE195920 gene data set was presented in three groups: the control groups (n = 3), the low fluoride exposure group (n = 3, U87 glial cells treated with 0.095 μg/mL fluoride for 10 days), and the high fluoride exposure group (n = 3, U87 glial cells treated with 0.22 μg/mL fluoride for 10 days). After that, differentially expressed genes between the control group and the fluoride-exposed group were selected based on the “limma” package. The cut-off value was set at *p* < 0.05 and |logFC (fold change)| ≥ 1 [65]. The original data were preprocessed by background adjustment, normalization, and log2 conversion. The FerrDb database (http://www.zhounan.org/ferrdb, accessed on 3 April 2022) is a manually collected and managed database consisting of ferroptosis-related markers, regulatory factors, and ferroptosis-related diseases [66].

### 4.2. Analysis of Differentially Expressed Genes in Samples

Volcano mapping was displayed using the enhanced volcano package (https://www.bioconductor.org/packages/release/bioc/vignettes/EnhancedVolcano/inst/doc/EnhancedVolcano.html, accessed on 3 April 2022). Heat mapping was conducted through the R software package pheatmap (https://cran.r-project.org/web/packages/pheatmap/index.html, accessed on 3 April 2022) display.

### 4.3. Identification of DEGs Related to Ferroptosis and Venn

Ferroptosis-related genes were downloaded from the FerrDb database and intersected with differentially expressed genes (DEGs). The Venn graph was plotted by http://www.bioinformatics.com.cn (accessed on 4 April 2022), a free online platform for data analysis and visualization.

### 4.4. Gene Ontology (GO) Enrichment Analysis of Ferroptosis-Related DEGs Genes and Kyoto Gene Encyclopedia and Genome Path Analysis

The function of ferroptosis-related DEGs was analyzed by gene ontology. Gene ontology function enrichment analysis included molecular function analysis (MF), biological process analysis (BP), and cell component analysis (CC). Analysis of the Kyoto Encyclopedia of Genes and Genomes (KEGG) was used to identify important pathways for gene enrichment. GO analysis and KEGG pathway analysis were performed using the cluster profile package [67] and the pathview package [68]. A *p* value < 0.05 was considered to be a statistically significant enrichment.

### 4.5. Protein–Protein Interaction (PPI) Network Analysis and Hub Gene Screening

STRING database (https://www.string-db.org/, accessed on 5 April 2022) is an online tool for analyzing protein–protein interaction. It can also be used to obtain unique, extensive, experimentally confirmed, and predictive interaction information. The comprehensive score shows the interaction between two proteins [69]. Relying on the STRING database, the PPI network of ferroptosis-related DEGs was constructed to predict the interaction between gene-encoded proteins, which may play an important role in the pathogenesis of fluorosis. In this study, only interaction pairs with a PPI composite score greater than 0.4 were selected as significant. Cytoscape software (version 3.9.1) was used to analyze the PPI network, and the Maximal Clique Centrality (MCC) [70] algorithm was used to screen the hub gene.

### 4.6. Screening of Potential Pharmacological Targets

The connectivity map (CMap) contains data on gene expression profile changes caused by 33,609 small-molecule compounds acting on multiple cell lines, representing the largest repository of compound-induced gene expression profiles, including the number of available compounds and the type of experimental conditions [71]. Further, the CMap query provides a similarity measure between upregulated or downregulated genes and disturbance-induced genes in the database [72], which can be used to compare the similarity between drug-induced gene profiles and gene expression profiles. Based on the nonparametric similarity measure of weighted Kolmogorov–Smirnov (KS) enrichment statistics, a connectivity score of −100 to 100 was obtained, where a score greater than 0 indicates that the changes caused by uploaded genes and compounds are similar, whereas score less than 0 indicates that the compound is opposite to the change of the uploaded gene; that is, the compound may have a therapeutic effect on the disease. The connectivity score of <−80 for small-molecule compounds was regarded as a promising prediction result [73].

### 4.7. Validation of Potential Therapeutics against CTD Database

The Comparative Toxicogenomics Database (CTD, http://ctdbase.org/, accessed on 5 April 2022) integrates a large number of data on the interaction between chemical substances, genes, functional phenotypes, and diseases, which provides great convenience for the study of disease-related environmental exposure factors and the potential mechanism of drugs [74]. In the CTD database system, the keyword used was “chemicals” and gene interactions of 10 potential therapeutic drugs were searched.

### 4.8. Immunohistochemical Verification of Overlapping Proteins

The expression of four overlapping proteins in skeletal muscle tissue and bone marrow tissue was verified by immunohistochemistry from the human protein map database (HPA, https://www.proteinatlas.org/, accessed on 5 April 2022) [75].

### 4.9. Molecular Docking

Two-dimensional structures of small-molecule ligands were retrieved from the PubChem (https://pubchem.ncbi.nlm.nih.gov/, accessed on 5 April 2022) database. In addition to that, two-dimensional structures of protein receptors were retrieved from Uniprot (https://www.uniprot.org/, accessed on 6 August 2022) and RCSB PDB (https://www.rcsb.org/, accessed on 6 August 2022) databases. PyMol software (version 2.5.3) was used to remove water molecules and original ligands, and pdbqt format conversion was performed. AutoDock Vina was used for molecular docking to assess the binding energy between ligands and receptors. Here, we noted that the smaller the binding energy, the more stable the docking module. Thus, a binding energy of <0 kcal/mol indicates that the ligand can bind to the receptor, whereas the binding energy of <-5.0 kcal/mol was considered suitable binding between receptor and ligand [76]. The interactions between receptors and ligands were analyzed by the Discovery Studio software (version 2019).

### 4.10. Molecular Dynamics Simulation and Post-Dynamic Analysis

GROMACS software (version 2020.4, Berendsen Lab, Gottingen County, Lower Saxony, GER) was used to simulate the molecular dynamics (MD) of small molecular prediction drug–protein docking complexes [77]. AMBER front field and AMBER99SB-ILDN force field were used to generate topological files for small molecules to predict drugs and proteins, respectively. The system is neutralized with NaCl counterion. Before MD simulation, it was necessary to minimize the complex by 1000 steps, and balance it by running NVT and NPT 100 ps, and then conduct MD simulation of 50 ns for each system under periodic boundary conditions at 310 K temperature and 1.0 bar pressure [78]. Using periodic boundary conditions, MD simulation was carried out in a triclinic box filled with TIP3 water molecules. The gmx rms and gmx rmsf programs were used to calculate root mean square deviation (RMSD) and root mean square fluctuation (RMSF), respectively; radius of gyration (Rg) was calculated by the gmx gyrate program. Gmx sasa was used to calculate the Solvent accessible surface area (SASA).

## 5. Conclusions

This study revealed the potential role of ferroptosis-related genes in fluorosis by involving bioinformatics methods and remarkably found that Celastrol and LDN-193189 could alleviate fluorosis symptoms by affecting ferroptosis-related genes, suggesting that both may be effective drug candidates for the treatment of fluorosis. The discovery of new targeted drugs could help alleviate the symptoms and late complications of fluorosis. Moreover, there could be a reduction in the disease burden and also an improvement in the quality of life of fluorosis patients. This will eventually provide a new theoretical basis for exploring treatment strategies. Furthermore, future drug validation and clinical trials will help in the discovery of new drugs to treat fluorosis in a timely and effective manner.

## Figures and Tables

**Table 1:** The 35 overlapping genes and their role in ferroptosis.

Type	Genes
Driver	G6PD, PIK3CA, SCP2, ACSL4, KRAS, GOT1, HMOX1, DPP4, CHAC1, ELAVL1, HIF1A, HMGB1, ATF3, LONP1, GCLC, IL6
Suppressor	PIK3CA, HMOX1, HIF1A, MT1G, FTMT, SCD, ACSL3, SESN2, JUN, CA9, LAMP2, ZFP36, CAV1, RRM2
Marker	ATF3, HMOX1, CHAC1, ELAVL1, HMGB1, SESN2, DUSP1, LOC284561, TXNIP, TRIB3, SNORA16A, IL6, CXCL2, HSD17B11, SLC2A14, RRM2

**Table 2:** CMap prediction of the top 10 drugs for fluorosis treatment.

Score	Name	Description
−99.93	Celastrol	Anti-inflammatory
−99.93	LDN-193189	Serine/threonine kinase inhibitor
−99.89	XPNPEP1	Methionyl aminopeptidase
−99.76	GNB5	WD repeat domain containing
−99.75	Importazole	Importin-beta transport receptor inhibitor
−99.74	LDHB	-
−99.66	NUAK1	NuaK subfamily
−99.63	IFNG	Interferons
−99.54	IFNB1	Interferons
−99.50	YWHAH	-

## Data Availability

Data sets of this study are available from GEO (https://www.ncbi.nlm.nih.gov/geo/, accessed on 1 August 2022).

## Supplementary Materials

The following supporting information can be downloaded at: https://www.mdpi.com/article/10.3390/ijms24044221/s1.
