# Supplementary material for: In Silico Analysis of Ferroptosis-Related Genes and Its Implication in Drug Prediction against Fluorosis
Source: Int J Mol Sci. 2023 Feb 20;24(4):4221. doi: 10.3390/ijms24044221 (PMC9961266; doi:10.3390/ijms24044221)
Supplement: Supplementary file 1 [file ijms-24-04221-s001.zip › ijms-2177003-supplementary.pdf]

Supplemental information

Table S1. Expression of 35 genes

Gene	Gene expression
G6PD	DOWN
TRIB3	DOWN
CXCL2	DOWN
ATF3	DOWN
ZFP36	DOWN
LOC284561	DOWN
CHAC1	DOWN
SESN2	DOWN
IL6	DOWN
FTMT	DOWN
SLC2A14	DOWN
HMOX1	DOWN
GCLC	DOWN
JUN	DOWN
DUSP1	DOWN
GOT1	DOWN
LONP1	DOWN
MT1G	DOWN
HIF1A	UP
ACSL3	UP
PIK3CA	UP
RRM2	UP
HSD17B11	UP
ELAVL1	UP
CAV1	UP
DPP4	UP
CA9	UP
LAMP2	UP
ACSL4	UP
KRAS	UP
SCD	UP
SCP2	UP
TXNIP	UP
SNORA16A	UP
HMGB1	UP

Table S2. Detail drawing of molecular docking site

Ligand	Receptor Protein	Free energy	Number of hydrogen bonds	Amino acids involved in H-bonding
	IL6	11 kcal/mol	2	ALA-59/GLU-173
Celastrol	JUN	-6.8 kcal/mol	2	THR-286/ ASN-291
	HMOX1	-8.5 kcal/mol	1	LYS-86
LDN-193189	HMOX1	--	1	SER A:14

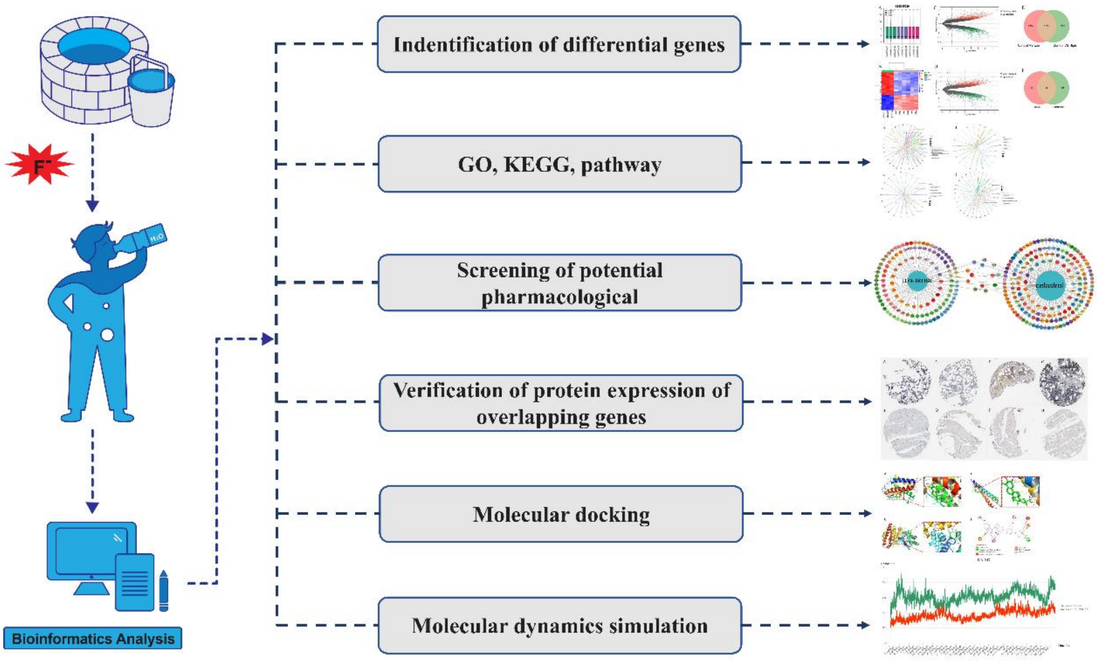

Flowchart S1. Flowchart of this manuscript. The flow chart shows the analysis process and main results of this study.
